# Superficial Keratectomy Alone versus in Combination with Amniotic Membrane Transplantation in Aniridia-Associated Keratopathy and a Short-Term Clinical Outcome

**DOI:** 10.3390/jcm13113258

**Published:** 2024-05-31

**Authors:** Bogumił Wowra, Marzena Wysocka-Kosmulska, Dariusz Dobrowolski, Edward Wylęgała

**Affiliations:** 1 Chair and Clinical Department of Ophthalmology, Faculty of Medical Sciences in Zabrze, Medical University of Silesia, 40-760 Katowice, Poland; m.wysocka554@gmail.com (M.W.-K.); dardobmd@wp.pl (D.D.); wylegala@gmail.com (E.W.); 2 Department of Ophthalmology, District Railway Hospital, 65 Panewnicka Street, 40-760 Katowice, Poland; 3 Department of Ophthalmology, St. Barbara Hospital, 41-200 Sosnowiec, Poland

**Keywords:** aniridia, keratopathy, superficial keratectomy, amniotic membrane transplantation, corneal neovascularization

## Abstract

**Background/Objectives**: Aniridia-associated keratopathy (AAK) is a potentially vision-threatening pathology in congenital aniridia, for which both the underlying etiopathogenesis and effective treatment remain unclear. **Methods**:This prospective study was conducted to assess and compare the short-term outcome after superficial keratectomy (SK) alone or in a combination with an amniotic membrane transplantation (AMT). Here, 76 eyes were enrolled in 76 patients with grade 4 AAK. In all eyes, in order to assess preoperatively the efficiency of the limbal epithelial stem cells (LESC), the presence of corneal epithelial cells in confocal microscopy was established. The analyses included: best corrected visual acuity (BCVA), the stage of AAK and the number of corneal quadrants involved in corneal neovascularization (CNV). **Results**: Six months after surgery, the mean BCVA was 0.05 and ranged from 0.002 up to 0.1 in both groups. Improvement in BCVA occurred in 94.29% patients when *SK alone* was performed, and in 92.68% when in combination with AMT. There were no statistically significant differences in the effect of therapy depending on the type of surgery, regarding BCVA, stage of AAK and the number of quadrants with CNV. **Conclusions**: SK alone is an effective procedure in short outcomes limited to six months for advanced AAK in association with LESC partial efficiency.

## 1. Introduction

Aniridia is a complex disease in which one of the basic features of changes in the eyeball is aniridia-associated keratopathy (AAK). The latter threatens the vision in these patients potentially the most. Nevertheless, these are not the only issues that affect these patients. Other diseases accompanying aniridia, which appear along with the child’s development, are nystagmus, dry eye disease, cataract, glaucoma, and disorders in the structure of the posterior pole of the eyeball, including the macular area and the optic nerve [1,2,3,4,5]. Aniridia is an inherited disease that has been quite well defined, and the basic defect is the mutation of the *PAX6* gene [6,7,8,9].

Pathological changes in patients with aniridia deteriorate over time, and they can occur at any time during the human lifespan. AAK usually starts within the first decade of life and progresses in young adults [9]. Therefore, it is crucial to diagnose it from early childhood as well as to identify and detect the progression of changes in individual tissues [10]. Regardless of the importance, it has not been possible to define unequivocally the pathogenesis of aniridia-associated keratopathy (AAK). Recent studies demonstrate additional changes that may be crucial in the etiopathogenesis of AAK, including morphological changes in the limbal niche, corneal nerves, corneal epithelium, and inflammatory cells [6,11,12,13,14,15,16,17]. Hence, it results in a decrease in visual acuity, mainly because of the malfunction of corneal epithelial stem cells located on the border of the cornea and sclera. The classification of aniridic keratopathy determines the different stages of the disease. According to the previously published scale by Lagali et al. [6], grade 0 of AAK indicates an intact limbus. The initial stages of AAK are ingrowth of the conjunctival epithelium onto the cornea just over the limbal border and displacement of phenotypically normal corneal epithelial cells (grade 1). In the next phase of keratopathy, the vascularized conjunctival tissue involves the mid-peripheral cornea (grade 3). In the most advanced phase (grade 4), in the entire cornea, additional to the conjunctival invasion and surface vascularization, the chronic inflammatory process with sub-epithelial fibrosis appears, resulting in massive pannus formation. 

There is no effective method of counteracting the development of these pathological changes to have been developed. In addition, we currently do not have any sufficient therapeutic practice to treat these disorders. Keratoplasty in the case of aniridia has a high risk of failure due to the concomitant limbal epithelial stem cells’ deficiency (LSCD) [18,19,20]. For this reason, one of the solutions that can temporarily improve the quality of vision in these patients is superficial keratectomy (SK) with a removal of pathological epithelium and subepithelial fibrosis [21,22,23,24]. The qualification for the procedure depends on whether the patients have a population of corneal epithelial cells, despite exhibiting the invasion of the conjunctival epithelium onto the cornea [12,15,25]. This is a prerequisite for the procedure to be successful, because in the event of complete limbal dysfunction, conjunctival tissue will recur almost immediately. Another temporarily effective method of treating these patients is amniotic membrane transplantation (AMT) [21,26,27]. The amniotic membrane, containing growth factors, cytokines, and other particles, has a positive effect on healing processes. Therefore, it promotes the migration and proliferation of the corneal epithelium cells, and inhibits corneal neovascularization (CNV) and scar tissue formation. Furthermore, its immunomodulatory and anti-inflammatory effects have been reported [28,29,30,31,32].

The paper represents the results of our experiment with corneal pannus removal in the most advanced grade of AAK. The aim of this study was to compare the short-term outcome of two surgical methods for the treatment of AAK in the advanced stage. These procedures are SK performed either solely or concomitantly with AMT. We hypothesized that the combination of both procedures may result in similar outcome versus SK implemented alone. This study will evaluate the necessity of performing additional AMT simultaneously with SK in patients with advanced AAK regarding the primary effect. 

## 2. Materials and Methods

### 2.1. Study Population 

The study population (*n* = 76) comprised adult patients over 18 years of age with grade 4 aniridia-associated keratopathy (AAK) accompanied by subepithelial fibrosis. The procedures were performed in the Department of Ophthalmology, District Railway Hospital (Katowice, Poland). The study was conducted from 2010 to 2023. All patients provided written informed consent prior to participation in the study. The study was achieved in accordance with the guidelines of the Helsinki Declaration and approval was obtained from the Silesian Medical Bord’s Bioethical Commission (Consent no. KNW/0022/KB1/35/14). Patients’ medical records were accessed to obtain applicable information (age, gender, BCVA, grade of AAK, and the number of quadrants involved with CNV). Characteristic features of the study population are shown in Table 1. 

### 2.2. Study Design

Here, 76 eyes were enrolled, belonging to 76 patients; all patients had low visual acuity of less than 0.1 on Snellen charts. 

Patients were randomly divided into two groups with a distribution of variables (age and gender). The first case group (A; *n* = 41) of patients underwent simultaneous surgery, with superficial keratectomy (SK) together with amniotic membrane transplantation (AMT), and in the second control group (B; *n* = 35), SK alone was performed.

The criterion for inclusion in the study was the patient’s consent, the presence of vascularized tissue in the central 6 mm of the cornea with accompanying subepithelial fibrosis, and the presence of the corneal epithelial cells in confocal microscopy (HRT 3, Heidelberg Engineering GmbH, Heidelberg, Germany). The factors excluded from the study were the inflammatory process on the surface of the eye, active conjunctival neovascularization, deep stroma neovascularization, unstable glaucoma requiring treatment intensification, including filtration procedures, and severe dry eye syndrome with inflammatory complications within the tissues, with epithelial defects or corneal ulcerations. In the qualifying examination, slit lamp microscopy allowed us to assess the stage of conjunctivalized and vascularized cornea, as well as the number of corneal quadrants occupied by vessels. 

### 2.3. Surgical and Postoperative Treatment

Procedures were performed under local anesthesia after the administration of Alcaine drops (Alcon, Fort Worth, TX, USA). Within the conjunctival tissue, approximately 2 mm from the corneal limbus, a pocket was formed, and the fibrous tissue was detached from the corneal surface with an ophthalmic spatula. The progress of the procedure was monitored intraoperatively with the use of OCT built into the operating microscope (Zeiss Artevo 800, Carl Zeiss Meditec AG, Jena, Germany). Therefore, it was possible to control the depth at which the pannus was detached from the conjunctival pocket with circular movements over the entire surface of the cornea. The procedure was carried out reaching the border of the limbus, then the tissue was cut with Vannas scissors, revealing the corneal stroma. The tissue was sent for a histopathological examination. Amniotic membranes were collected from healthy donors after cesarean sections, with the patients’ consent, and standardly prepared by the Homograft Tissue Bank in Zabrze. The amniotic membrane patch was fixed with a running suture to the conjunctiva facing with the stromal side the entire cornea. After the procedure of superficial keratopathy, in the first case group, AMT and the bandage contact lens were applied, and in the second control group, contact lens alone was used. 

Antibiotic and steroid drops without preservatives were used, administered every two hours in the first three days, and then 4 times a day until the cornea was reepithelialized. The treatments were terminated after 14 days. The bandage contact lens was removed in case of a complete reepithelialization. Subsequently, the patients continued to use only unpreserved artificial tears containing at least hyaluronic acid.

### 2.4. Follow-Up Data Collection

Check-ups were performed every 3 months. Each visit was accompanied by a complete ocular examination, including best corrected visual acuity (BCVA), intraocular pressure assessment, slit lamp biomicroscopy, photo slit lamp imaging, laser scanning in vivo confocal microscopy (HRT 3, Heidelberg Engineering GmbH, Heidelberg, Germany) and optical coherence tomography (OCT) (SS OCT; CASIA2 OCT; Tomey, Nagoya, Japan). In the slit lamp microscopy epithelial regeneration, conjunctival vascularization and subepithelial fibrosis status were assessed. These tests allowed us to evaluate the degree of AAK and the number of affected corneal quadrants with corneal neovascularization (CNV). Data were recorded to analyze following variables: BCVA, AAK stage and the number of corneal quadrants with CNV. To assess BCVA, the Snellen chart was used; for hand motion (HM) and counting fingers (CF) visual acuity, respectively, 0.002 and 0.005 values were assigned. The degree of AAK was rated on the basis of a scale proposed by Lagali et al. [6]. To calculate the quadrants of the cornea affected by CNV, in the slit lamp examination, we divided the cornea into 4 equal parts using two perpendicular lines that extended from 9 to 3 and from 12 to 6, respectively, according to the clockwise division of the corneal circumference. The primary outcome was assessed within an observation period that lasted 6 months. 

### 2.5. Statistical Analysis

Statistical analyses were performed with Statistica v.13.1 package. Data were expressed as means ± standard deviation (SD). The Shapiro–Wilk test was used to verify the normality of the distribution of the analyzed variables. To test the significance of differences, the parametric Student’s *t*-test for independent samples, the non-parametric Wilkoxon pair order test for dependent samples, and the U Mann–Whitney test for independent samples were assessed. Spearman’s non-parametric rank correlation coefficient was used to analyze associations. For variables measured on the rank and nominal scales, frequencies and structure indicator values (percentages) were calculated. To verify whether there are connections between qualitative features, Pearson’s χ2 independence tests with the NW correction and Yates’ χ2 tests were applied. A value of *p* < 0.05 was considered statistically significant.

## 3. Results

### 3.1. Characteristics for Study Group

A total of 76 eyes from 76 patients with AAK were included in this study. The mean age was 45 ± 15 years (range: 22–74 years); 41 (54%) patients were females and 35 (46%) were males. The differences related to age and gender between the groups did not show any statistical significance (*p* value > 0.05). The population was randomly divided into two groups: either the case group (A), where AMT was performed together with SK, or the control group (B), in which SK alone was carried out. Preoperatively, all the subjects had stage 4 of keratopathy and four corneal quadrants occupied by CNV. All the characteristics are shown in Table 1. 

### 3.2. Follow-Up

#### 3.2.1. Visual Acuity Outcome 

**Figure 1:**
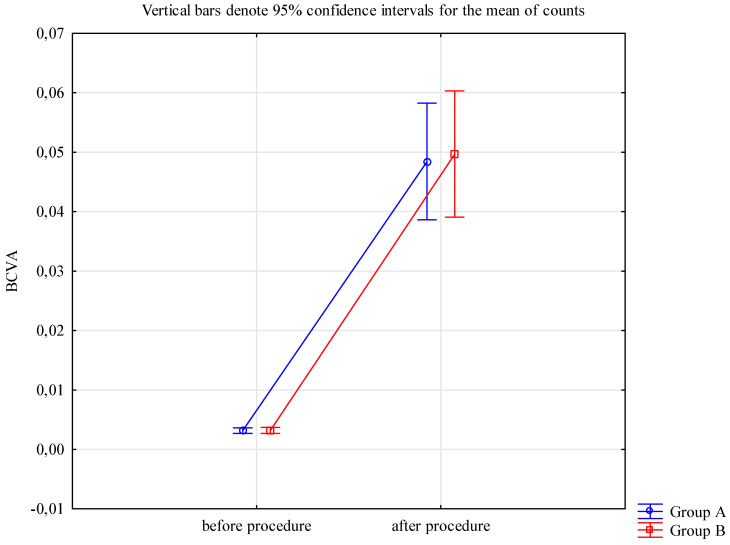
The mean values of BCVA before and after the procedures in both groups significantly improved. Group A (case group)—SK with AMT (*n* = 41); Group B (control group)—SK alone (*n* = 35); BCVA—best corrected visual acuity. Vertical bars denote 95% confidence intervals for the mean of counts.

Preoperatively, the mean BCVA in both groups was 0.0032, and ranged from 0.002 (hand motions; HM) to 0.005 (counting fingers; CF). Low BCVA values were attributed to stage 4 of AAK, where the central cornea is covered with the pannus. Six months after surgery, the mean BCVA was 0.05 and ranged from 0.002 to 0.1 in both groups. The mean difference between BCVA before and after procedure in both groups was 0.05, ranging from 0.000 to 0.095 (Table 2). Postoperatively, the improvement in visual acuity was limited due to the comorbidities in aniridia (coexisting foveal and optic nerve hypoplasia). The analysis of the results for both the study (A) and control (B) groups allowed for the detection of a significantly statistic improvement in BCVA in patients six months after surgery compared to BCVA before procedure (*p* value < 0.05; Wilcoxon pair order test). These results are confirmed in Figure 1. Most importantly, there was no statistically significant difference in the effect of therapy depending on the group regarding BCVA (*p* value = 0.86; U Mann–Whitney test). 

Additionally, BCVA results were ranked in order to verify in the subsequent analyzes whether the BCVA results before and after therapy were related to the groups (Yates’s χ2 tests for 2 × 2 tables and Pearson’s χ2 tests with the NW correction) (Table 3). The analyses demonstrate that the groups were not statistically significantly associated with BCVA before and after therapy (*p*-value > 0.05). Preoperatively, BCVA was 0.002 (HM) in the majority of subjects in the case (A) and the control (B) groups—60% and 61%, respectively. In most cases—92.68% in the case (A) group and 94.29% in the control (B) group—BCVA 6 months after the procedure improved, and was within the range from 0.05 to 0.1; 7.32% of patients in the case group and 8.47% of patients in the control group achieved a BCVA of 0.1.

Subsequent calculations revealed that in the group that underwent the SK procedure alone, an improvement of BCVA occurred in 94.29% patients, while in the group of patients who underwent SK and AMT, this value was 92.68%. The improvement in BCVA after therapy in both groups was not statistically significantly related to the group (*p*-value = 0.85). In the case of both groups, significant differences were found between the percentage of recipients who had improved results regarding BCVA and those without improvement. Based on the significance test for the structure index (Student’s t-statistics), it was found that in both groups, the percentage of people with improved results was statistically significant (*p*-value < 0.00001). There were no differences in the percentages of patients in both groups whose results improved (*p*-value = 0.78).

Subsequent analyses verified whether age or gender were related to the effect of therapy (ΔBCVA), for all subjects and all groups. Interestingly, it was found that the effect of therapy decreased with increasing age (Spearman’s rank order correlation; R = −0.38; *p*-value = 0.0008 for all subjects). Moreover, the analysis of the variables gave rise to the result that the effect of therapy in women was statistically notably higher than it was in men (U Mann–Whitney Test; *p*-value = 0.0013 for all recipients).

#### 3.2.2. Slit Lamp Examination Outcomes

In the qualification exam, AAK in stage 4 was observed in 100% of the subjects in both groups (Table 4). Then, 6 months after the procedure, in most cases the degree of AAK decreased to “O”. This result was observed in over 70% of cases; precisely, 29 subjects (70.73%) from the study group A where SK was performed in a combination with AMT, and 25 subjects (71.43%) from the control group where only SK was performed. There was no evidence that the type of procedure significantly differentiated the results of the analyzed variables with regard to the stage of AAK (*p*-value > 0.05).

When the number of corneal quadrants involved in conjunctival vessel invasion (CNV) was assessed in the examination before the procedure, all four quadrants were found to be involved in all study groups (Table 5). At the end point of the study, the majority of subjects showed the presence of CNV in one quadrant—respectively, 24 subjects (58.54%) in the case group, and 21 subjects (60%) in the control group. Comparably to AAK, the results for the number of quadrants with CNV were not statistically significantly associated with patient groups (*p*-value > 0.05).

Moreover, there was no evidence that the type of procedure significantly differentiated the results for either of the analyzed variables according to the U Mann–Whitney test (*p*-value = 0.94 and *p*-value = 0.92, respectively, for AAK staging and quadrants with CNV).

Epithelial healing time was estimated after 4 weeks when the sutures of amniotic membrane were removed, and then uncompleted epithelialization was recognized. The only post-operative complication was prolonged time of re-epithelialization.

IVCM was performed to estimate only the corneal origin of the epithelium. This would be helpful in confirming the remaining function of stem cells. We did not qualify the level of stem cell insufficiency in IVCM because of a limited ability to check the whole limbal area in cases of limbal stem cell damage.

**Figure 2:**
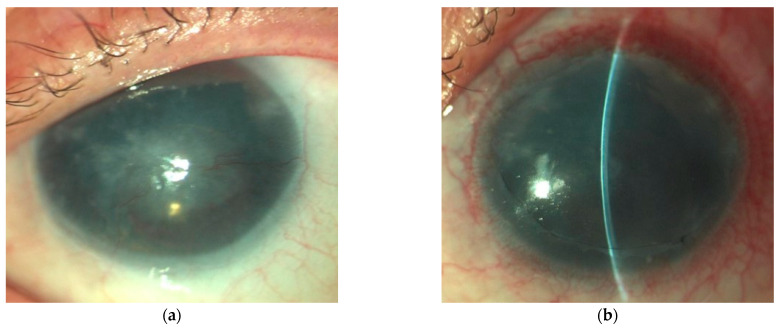
The image shows a slit lamp photo of the patient included in the control group, where superficial keratectomy (SK) alone was performed. (**a**) Stage 4 of aniridia-associated keratopathy (AAK) with a visible fibrovascular scar in the central part of the cornea before SK. (**b**) After removal of the pannus at the first follow-up visit with decreased stage of AAK, none of the quadrants were affected by corneal neovascularization (CNV), and complete corneal reepithelialization with smooth epithelium was presented.

**Figure 3:**
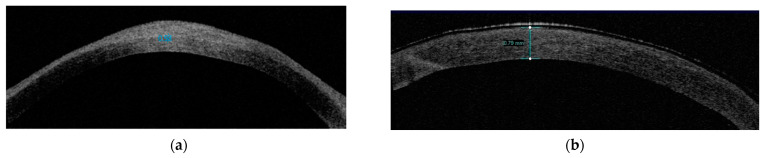
Anterior segment-optical coherence tomography. (**a**) The image shows a hyper-reflective pannus covering the central cornea (with a limitation of measurements). (**b**) The post-SK photo reveals the total corneal thickness after the removal of the corneal pannus (with a limitation of measurements).

**Figure 4:**
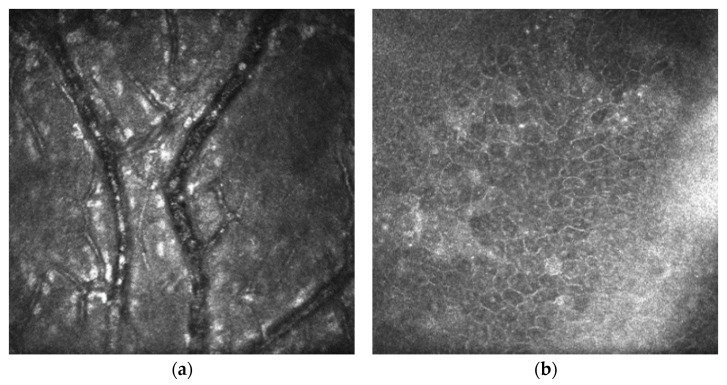
Laser scanning confocal microscopy before and 6 months after surgery (SK alone). (**a**) Corneal neovascularization (CNV) visible at the level of the epithelium before SK. (**b**) The wing and basal epithelial cells with bright activated nuclei after SK.

Example photos of a 49-year-old man before and after the SK surgery are presented (Figure 2, Figure 3 and Figure 4).

## 4. Discussion

The findings of this study confirm the hypothesis that SK alone has a similar outcome as when in a combination with AMT. When comparing study groups, regarding the improvement in BCVA, and the reductions in the AAK stage and the number of vascularized corneal quadrants (CNV), the results of treatments in the 6-month period were similar. Additionally, both types of procedures, either SK alone or in relation with AMT, are here proven to be effective, with similar results. A total of 76 subjects were included in this study. BCVA was improved in over 90% of cases—respectively, 94% and 93%—when SK alone or SK combined with AMT were used. Here, 46% of subjects achieved a final BCVA within the range of 0.05 up to less than 0.1. In over 70% of cases, the stage of AAK decreased from 4 to 0, and nearly 60% of eyes showed reduced vascularization from 4 to 1 in the corneal quadrant; all these results did not differ between the groups.

To our best knowledge, no previous studies have evaluated the effectiveness of treatment with these procedures in congenital aniridia, or compared their effectiveness. This is also the first study with such a large study group. The reason that neither SK nor SK in combination with AMT have been introduced as a standard practice in the treatment of AAK is probably due to the pathogenesis of AAK, and so it will be appropriate to proceed by addressing the cause of the pathology. Additionally, congenital aniridia is a rare disease, and the possibility of creating a large study group is limited. This is not a wide group of patients with limbal stem cells deficiency (LSCD), which also includes patients with congenital aniridia. Matsumura et al. [23] showed the long-term effect of SK in patients with partial LSCD. As mentioned before, the inclusion criterion for our study was the presence of corneal epithelial cells in confocal microscopy as proof of at least the partial efficiency of limbal stem cells. Matsumura et al. included 51 eyes without dense fibrovascular tissue (grade 2 of LSCD), which result is different from that of our study group (grade 4 of AAK). Furthermore, the visual acuity improved or remained unchanged postoperatively, with a mean follow-up period of 26.3 months. The final success rate was 84%. Interestingly, regarding the mean time of epithelialization, recurrent rate, and the mean time of recurrence, no statistically significant difference was observed between eyes with SK and when additional AMT was performed.

The amniotic membrane, containing numerous growth factors, cytokines, and stem cells, has regenerative properties for the corneal epithelium, as well as anti-inflammatory, immunomodulating, and angiomodulating properties, and it inhibits scar formation [28,29,30,31,32]. All these elements are beneficial for AAK, addressing its pathogenesis. Nevertheless, our study did not prove the superiority of SK combined with AMT over SK alone. This may be related to the limited time for which the amniotic membrane can affect the healing of tissue, or to the fact that AMT does not sufficiently address the etiology of keratopathy in congenital aniridia. Moreover, despite the well-known regenerative features of the amniotic membrane for corneal epithelial cells, in this study, measurements of the re-epithelialization process have not been precisely described. 

Studies in patients with congenital aniridia using either SK or AMT are very limited. However, a few studies have been conducted on this group of patients. In a retrospective cohort study, Yazdanpanah et al. [21] summarized the treatment of 92 eyes with congenital aniridia. It was determined that among subjects, four people underwent SK and three underwent AMT. However, no effect of these treatments was noted. Shortt et al. [22] cultured ex vivo limbal epithelial stem cells (LESC) on human amniotic membrane, and these were transplanted with the amniotic membrane to the recipient eyes after SK in patients with LSCD. Among this study group, three patients suffered from AAK. The implementation of this treatment allowed for an improvement in visual acuity from CF up to a maximum of 0.2 in selected patients, similarly to in our study. Jacobson et al. [24] mentioned that SK with AMT may be used in addressing early scarring. In contrast, we implemented both types of surgeries in patients with advanced scar tissue formation. López-García et al. [26] studied 14 eyes of patients with congenital aniridia and moderate limbal deficiency that were treated with AMT. BCVA improved by an average of 0.3 during the 24-month follow-up period, as well as corneal clarity and peripheral neovascularization during the initial follow-up period. On the contrary, we did not observe AMT superiority over SK alone in BCVA or CNV withdrawal outcome. However, 9 months after the observation, López-García et al. described metaplasia in epithelial cells and their return to an initial state. Tseng et al. [27] applied AMT in patients with LSCD, among whom three had eyes with congenital aniridia. After AMT, the corneal surface became smooth, stable, and transparent enough to regain BCVA before the surgery. 

Interestingly, a statistically notable difference was found in postoperative BCVA, as women and younger people had better results. Regarding age, Ang et al. [33] showed in a study including 45% people with AAK that younger age was associated with increased risk of rejection after stem cell transplantation. Therefore, performing an alternative SK procedure may be beneficial in young adults.

It is currently argued that the appropriate approach to the treatment of AAK is a compound of the multiple therapeutic options. In the past, AAK has most commonly been treated with penetrating or lamellar keratoplasty alone; nevertheless, these procedures entail a high risk due to their postoperative complications and treatment failure [18,19,20]. Other surgically available alternatives include limbal stem cell transplantation (LSCT) and Boston type 1 Keratoprosthesis (KPro) implantation [18,34,35]. Nevertheless, non-negligible disadvantages of these therapeutic options are connected with the lifelong pharmacological suppression of the immune system due to the prevention of rejection [36,37]. 

In this study, we demonstrated that an alternative treatment for congenital aniridia is SK alone or SK combined with AMT. Although SK itself does not address the cause of AAK, when performed alone, it appears to be a safe and effective procedure that can temporarily improve vision and the state of the ocular surface. We propose SK alone as an alternative procedure for advanced AAK in association with the pannus. The effectiveness of the procedure is based on the mechanical removal of the superficial corneal opaqueness, which is caused by a growth of blood vessels and subepithelial fibrous connective tissue. This allows for the re-epithelialization of the denuded corneal surface, and has a positive effect on the refractive error and visual acuity. However, a necessary condition for the formation of new corneal epithelial cells and the smoothing of the surface, with a reduction in epithelial irregularities, is the at least partial efficiency of the limbal epithelial stem cells (LESC). Unfortunately, limbal stem cell abnormalities affect the majority of patients with congenital aniridia. Therefore, the inclusion criterion for safe SK is the presence of corneal epithelial cells, which proves the partial efficiency of LESC; otherwise, superficial keratopathy would be an ineffective procedure.

## Figures and Tables

**Table 1:** Characteristics of case and control groups before procedures.

Characteristics	Case Group Group A *n* = 41	Control Group Group B *n* = 35	*p*-Value
Age (years)	44.78 ± 14.71	45.86 ± 14.44	0.75
Females	22 (53.66%)	19 (54.29%)	0.86
Males	19 (46.34%)	16 (45.71%)	0.86
BVCA before procedure	0.0032 ± 0.0015	0.0032 ± 0.0015	1
Stage 4 of AAK	41 (100%)	35 (100%)	
CNV in 4 corneal quadrants	41 (100%)	35 (100%)	

**Table 2:** Comparison of BCVA at the baseline and 6 months after the intervention, and the difference in BCVA in both groups.

Characteristics	Estimates	Case Group (A) *n* = 41	Control Group (B) *n* = 35	Case Group (A) *p*-Value *	Control Group (B) *p*-Value *
BCVA before procedure	Mean ± standard deviation	0.0032 ± 0.0015	0.0032 ± 0.0015	<0.00001	<0.00001
	CI −95% CI 95%	0.0027 0.0037	0.0027 0.0036		
BCVA 6 months after procedure	Mean ± standard deviation	0.0484 ± 0.0314	0.0497 ± 0.0316	0.0036	0.0095
	CI −95% CI 95%	0.0385 0.0584	0.0388 0.0606		
∆BCVA	Mean ± standard deviation	0.0453 ± 0.0302	0.0465 ± 0.0304	0.0038	0.011
	CI −95% CI 95%	0.0357 0.0548	0.0360 0.0569		

Data presented as mean ± standard deviation (X ± SD), and CI—95% confidence interval of the mean (a range with an upper and lower number calculated from a sample). Case group (Group A)—SK with AMT (*n* = 41). Control group (Group B)—SK alone (*n* = 35). ∆BCVA—BCVA after—BCVA before procedure. SK—superficial keratopathy. AMT—amniotic membrane transplantation. BCVA—best corrected visual acuity. * The results of the Shapiro–Wilk test are reported at a significance level of 0.05.

**Table 3:** Ranked BCVA results and occurrence in both groups.

		Before the Procedure		6 Months after the Procedure	
Ranges	BCVA	Case Group (A) *n* = 41	Control Group (B) *n* = 35	Case Group (A) *n* = 41	Control Group (B) *n* = 35
1	HM (0.002)	25 (60.98%)	21 (60%)	3 (7.32%)	2 (5.71%)
2	CF (0.005)	16 (39.2%)	14 (40%)	6 (14.63%)	5 (14.29%)
3	0.01–0.049	0	0	10 (24.39%)	9 (25.71%)
4	0.05–0.99	0	0	19 (46.34%)	16 (45.71%)
5	0.1	0	0	3 (7.32%)	3 (8.57%)

Group A (case group)—SK with AMT (*n* = 41); Group B (control group)—SK alone (*n* = 35); SK—superficial keratopathy; AMT—amniotic membrane transplantation; BCVA—best corrected visual acuity.

**Table 4:** Presentation of the occurrence of subsequent stages of AAK in individual groups before and 6 months after procedure.

	Before the Procedure		6 Months after the Procedure	
Stage of AAK	Case Group (A) *n* = 41	Control Group (B) *n* = 35	Case Group (A) *n* = 41	Control Group (B) *n* = 35
0	0	0	29 (70.73%)	25 (71.43%)
1	0	0	3 (7.32%)	2 (5.71%)
2	0	0	9 (21.95%)	8 (22.86%)
3	0	0	3 (7.32%)	2 (5.71%)
4	41 (100%)	35 (100%)	0	0

Group A (case group)—SK with AMT (*n* = 41); Group B (control group)—SK alone (*n* = 35); SK—superficial keratopathy; AMT—amniotic membrane transplantation; AAK—aniridia-associated keratopathy.

**Table 5:** The results reveal the number of affected corneal quadrants before and after the procedure in both groups.

	Before the Procedure		6 Months after the Procedure	
The Number of Corneal Quadrants with CNV	Case Group (A) *n* = 41	Control Group (B) *n* = 35	Case Group (A) *n* = 41	Control Group (B) *n* = 35
0	**0**	**0**	2 (4.88%)	2 (5.71%)
1	0	0	24 (58.54%)	21 (60%)
2	0	0	14 (34.15%)	10 (28.57%)
3	0	0	1 (2.44%)	3 (5.71%)
4	41 (100%)	35 (100%)	0	0

Group A (case group)—SK with AMT (*n* = 41); Group B (control group)—SK alone (*n* = 35); SK—superficial keratopathy; AMT—amniotic membrane transplantation; CNV—corneal neovascularization.

## Data Availability

The data used to support the findings of this study are included in the article. The data will not be shared due to third-party rights and commercial confidentiality.
